# Nicotine Receptor Subtype-Specific Effects on Auditory Evoked Oscillations and Potentials

**DOI:** 10.1371/journal.pone.0039775

**Published:** 2012-07-20

**Authors:** Robert E. Featherstone, Jennifer M. Phillips, Tony Thieu, Richard S. Ehrlichman, Tobias B. Halene, Steven C. Leiser, Edward Christian, Edwin Johnson, Caryn Lerman, Steven J. Siegel

**Affiliations:** 1 Department of Psychiatry, University of Pennsylvania, Philadelphia, Pennsylvania, United States of America; 2 Department of Psychology, Mount St. Mary’s University, Emmitsburg, Maryland, United States of America; 3 AstraZeneca, Wilmington, Delaware, United States of America; Chiba University Center for Forensic Mental Health, Japan

## Abstract

**Background:**

Individuals with schizophrenia show increased smoking rates which may be due to a beneficial effect of nicotine on cognition and information processing. Decreased amplitude of the P50 and N100 auditory event-related potentials (ERPs) is observed in patients. Both measures show normalization following administration of nicotine. Recent studies identified an association between deficits in auditory evoked gamma oscillations and impaired information processing in schizophrenia, and there is evidence that nicotine normalizes gamma oscillations. Although the role of nicotine receptor subtypes in augmentation of ERPs has received some attention, less is known about how these receptor subtypes regulate the effect of nicotine on evoked gamma activity.

**Methodology/Principal Findings:**

We examined the effects of nicotine, the α7 nicotine receptor antagonist methyllycaconitine (MLA) the α4β4/α4β2 nicotine receptor antagonist dihydro-beta-erythroidine (DHβE), and the α4β2 agonist AZD3480 on P20 and N40 amplitude as well as baseline and event-related gamma oscillations in mice, using electrodes in hippocampal CA3. Nicotine increased P20 amplitude, while DHβE blocked nicotine-induced enhancements in P20 amplitude. Conversely, MLA did not alter P20 amplitude either when presented alone or with nicotine. Administration of the α4β2 specific agonist AZD3480 did not alter any aspect of P20 response, suggesting that DHβE blocks the effects of nicotine through a non-α4β2 receptor specific mechanism. Nicotine and AZD3480 reduced N40 amplitude, which was blocked by both DHβE and MLA. Finally, nicotine significantly increased event-related gamma, as did AZD3480, while DHβE but not MLA blocked the effect of nicotine on event-related gamma.

**Conclusions/Significance:**

These results support findings showing that nicotine-induced augmentation of P20 amplitude occurs via a DHβE sensitive mechanism, but suggests that this does not occur through activation of α4β2 receptors. Event-related gamma is strongly influenced by activation of α4β2, but not α7, receptor subtypes, while disruption of N40 amplitude requires the activation of multiple receptor subtypes.

## Introduction

Individuals with schizophrenia display changes in auditory event-related potentials (ERPs) including decreased amplitude of the P50 and N100 components and disrupted gating of the P50 [1], [2], [3], [4], and these are assumed to reflect deficits in elementary information processing. Nicotine has been shown to enhance P50 gating in people with schizophrenia and their first degree relatives [4], [5], [6], suggesting that nicotinic agents could be useful for the treatment of schizophrenia. Evidence from studies assessing pharmacological response, changes in response to parametric manipulations and response to novelty suggest that the mouse P20 and N40 are analogous to the human P50 and N100, respectively [7], [8], [9], [10], [11], [12], [13], [14], [15]. In particular numerous studies have demonstrated increases in mouse P20 amplitude following nicotine administration, as well as nicotine-induced decreases in mouse N40 amplitude [10], [11], [15]. Thus, rodent ERP methodologies have great potential for translational drug discovery in schizophrenia.

In addition to changes in the ERP, nicotine has been shown to enhance power in the gamma frequency range of the EEG [15], [16]. Gamma oscillations are thought to be generated in part by parvalbumin expressing GABAergic interneurons, a cell population that is disrupted in schizophrenia [17]. As such, gamma oscillations have been proposed as an important biomarker of the integrity of this cell population [17]. Numerous studies have demonstrated reduced or altered gamma power in schizophrenia and in physiologically relevant animal models of schizophrenia [17], [18], [19]. Increases in gamma power have been demonstrated during performance of cognitive tasks in control subjects, especially during attention and working memory [20], suggesting that enhanced gamma activity may serve as a mechanism through which nicotine influences schizophrenia symptomology and cognition.

Recent studies have attempted to identify the specific nicotinic acetylcholinergic receptor subtypes responsible for regulating the effects of nicotine on ERPs, and more recently, evoked gamma activity. At present the mechanism by which nicotine enhances P20 amplitude is not entirely clear, although both α7 and α4β2 nicotinic receptors have been implicated [21], [22], [23], [24], [25], [26], [27]. Transgenic mice lacking the β2 subunit show a typical nicotine-induced enhancement in P20 response to the first stimulus (S1) of a paired stimulus presentation but fail to show the normal nicotine-induced decrement in N40 S1 response [25], suggesting that the role of the α4β2 receptor in sensory gating primarily involves the β2 subunit and is limited to regulation of the N40, but not P20, ERP component. While the α7 receptor has been shown to influence P20 response, this appears to occur primarily through decreased amplitude of response to the second stimulus (S2) of a stimulus pair [26], with S1 being relatively unchanged. Much less is presently known about how nicotine influences gamma EEG. To resolve these questions, the present study examined the effects of nicotine, the α7 nicotinic acetylcholine receptor antagonist methyllycaconitine (MLA), the α4β4/α4β2 nicotinic acetylcholine receptor antagonist dihydro-beta-erythroidine (DHΒE) and the α4β2 receptor agonist AZD3480 on amplitude and gating of the P20 and N40 ERP components as well as baseline and evoked gamma oscillations in mice.

## Methods

### Animals

Thirteen male C57BL/6J mice were used for experiment 1 (nicotine antagonist study) and 9 additional mice were used for experiment 2 (AZD3480 study). All mice were obtained at 8 weeks of age from Jackson Laboratories (Bar Harbor, ME), and were assessed at 10 to 12 weeks of age. Mice were housed four to five per cage until electrode implantation and single-housed thereafter in a light- and temperature-controlled Association for Assessment and Accreditation of Laboratory Animal Care-accredited animal facility. Water and standard rodent chow were available *ad libitum*. Experiments were conducted at the University of Pennsylvania during the light phase between 9∶00 AM and 3∶00 PM. Mice were allowed at least one week to acclimate to the housing facility before starting any procedure. All protocols were performed in accordance with University Laboratory Animal Resources guidelines and were approved by the Institutional Animal Care and Use Committee at the University of Pennsylvania (803887).

### Drugs

Nicotine hydrogen tartrate salt (1.0 mg/kg), methyllycaconitine (MLA –10.0 mg/kg), and dihydro-beta-erythroidine (DHΒE - 2.0 mg/kg) (Sigma-Aldrich, St. Louis, MO, USA) were dissolved in 0.09% saline. Nicotine was administered intraperitoneally (I.P) at a volume of 0.1 ml. MLA and DHΒE were administered subcutaneously, also at a volume of 0.1 ml. All drug doses are expressed as drug base and were selected based on previous research on nicotine’s effects on ERPs [10], [11] and MLA and DHΒE’s ability to block behavioral effects of nicotine [28], [29], [30], [31]. AZD3480 (TC-1734) was obtained from AstraZeneca and was administered I.P. in a volume of 0.1 ml at a dose of 1 and 10 mg/kg. AZD3480 is a potent agonist of the nicotinic α4β2 receptor [32], [33], [34] that has shown efficacy for treatment of cognitive impairments across a broad class of disease conditions.

### Electrode Implantation

Unipolar recording electrodes were unilaterally placed in the CA3 hippocampal region under isoflurane anesthesia, (1.8 mm posterior, 2.65 mm lateral, and 2.75 mm deep relative to bregma) and referenced to the surface of the ipsilateral frontal hemisphere (0.8 mm anterior, 2.65 mm lateral and 1 mm deep, relative to bregma). Since the recording and reference electrodes are located far apart from one another, it is likely that activity recorded using this configuration extends far beyond the localized field generated within the CA3 region, and, therefore, as in human EEG recordings, reflects brain activity across a widespread area. For this reason, histological verification of electrode placement was not performed in the current study, although this has been reported elsewhere using the same surgical procedures [8]. ERPs recorded using this configuration are characteristically similar to human recordings from the Cz scalp location, as illustrated in a prior publication by our group [13], and show numerous pharmacological (amphetamine, ketamine, nicotine, etc.) and parametric responses (effects of ISI), similar to those observed in human EEG studies from Cz [11], [25], [35], [36], [37], [38]. Dental cement and ethyl cyanoacrylate (Elmers, Columbus, OH) were used to secure the electrode pedestal to the skull. Procedures were consistent with descriptions published elsewhere [8], [9], [10], [11], [13].

### Recording

For experiment 1, five days of ERP testing was conducted over a two week period with a minimum of 24 hours washout period between drug exposures and three recording sessions per test day. The first recording session of each test day was an acclimation trial followed by a baseline saline (vehicle) trial. The third testing session consisted of the injection of the test compound(s). On the first testing day, animals were acclimated to all handling and testing procedures and received nicotine (1 mg/kg). On test day two, animals received MLA (10 mg/kg), on test day three a combination treatment of MLA (10 mg/kg) followed 5 minutes later by nicotine (1 mg/kg). On the fourth day of testing, animals received an injection of DHβE (2 mg/kg) and on the fifth and final testing day animal received a combination treatment of DHβE (2 mg/kg) followed 5 minutes later by nicotine (1 mg/kg). For experiment 2 (AZD3480 study, ERPs were assessed over three successive days, with each day being separated by a 72 hour period. Mice were tested following saline vehicle, 1 mg/kg and 10 mg/kg on days 1, 4 and 7, respectively. Additionally, in order to assess potential carry over effects of drug treatment, baseline response was assessed prior to each test session.

#### ERP analysis

In both experiments ERPs were recorded following 50 stimulus presentations. Testing commenced five minutes after the last injection. Stimuli were generated by Micro1401 hardware and Spike2 software (Cambridge Electronic Design, Cambridge, UK) and were delivered through speakers attached to the cage top. Recordings were performed in 8 standard mouse cages, all of which were located within a Faraday cage. 50 pairs of white noise clicks (85 db, 10 ms in duration) were presented with a 9-s interstimulus interval against 70 dB of background noise. Waveforms for the ERP and gamma analyses were filtered between 1 and 500 Hz and baseline corrected at stimulus onset. Individual sweeps were rejected for movement artifact based on a criterion of 2 times the root mean squared amplitude per mouse. Average waves were created from 50-ms pre-stimulus to 200-ms post-stimulus. The P20 component was selected for each ERP by determining the maximum positive deflection between 10 and 30 ms (S1) and between 510 and 530 ms (S2). The N40 was selected by determining the maximum negative deflection between 25 to 60 ms (S1) and between 525 and 560 ms (S2) post-stimulus. Since the latency of the maximum deflection can differ greatly between animals, this method of quantification will produce values for P20 and N40 that are oftentimes higher than what appears in the grand average waveform, which tends to smooth out maximal differences. Sensory gating was analyzed by comparing the amplitude of S1 to the amplitude of S2, with successful sensory gating being defined as a statistically significant difference between S1 relative to S2, with S1 being greater than S2, and sensory gating failure being defined as a lack of statistically significant difference between S1 and S2. This was done separately for P20 and N40.

Event-related gamma (30 to 80 Hz) data were processed using EEGLAB (Schwartz Center for Computational Neuroscience). Epochs were extracted from between −199 and 399 msec relative to tone onset. Power was calculated using Morlet wavelets in 116 logarithmically spaced frequency bins between 4 and 120 Hz, with wavelet cycle numbers ranging from 2 to 10 [39]. Average post-stimulus (0 to 60 milliseconds) gamma power was calculated for each animal, and this served as the primary measure of gamma power for statistical analysis. Gamma was defined as being between the frequencies of 30 and 80 Hz.

### Analysis

Both the antagonist and AZD3480 experiments used a repeated within-subjects design in which all animals were exposed to each level of DRUG and each level of STIM (S1 and S2 components). Separate analyses were conducted for the P20, N40 and for event-related gamma The P20 and N40 data were analyzed using a 2 factor repeated measures ANOVA (DRUG by STIM), while gamma data were analyzed using a single factor repeated measures ANOVA (DRUG). In both experiments, repeated measures analyses of variance (ANOVAs) were performed on baseline data for each ERP component to rule out possible confounding effects of time and repeated testing. In the case of a significant main effect or interaction, Fisher’s LSD post-hoc tests were conducted. All tests were two-tailed. Results are significant at *p*≤0.05 unless otherwise noted.

## Results

### Event Related Potentials

**Figure 1:**
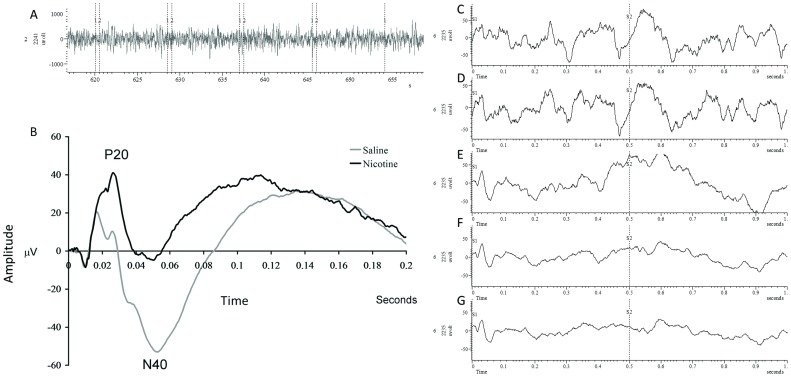
Effects of nicotine on the grand average waveform of the ERP. **A**) Raw EEG trace from an individual animal showing 4 stimulus pair presentations. Lines labeled 1 show S1, while lines labeled 2 show S2. **B**) Grand averages for auditory Event Related Potential are shown for the saline (gray) and nicotine (black) conditions. As in previous studies, nicotine significantly increased amplitude of the P20 component and reduced amplitude of the N40. These grand averages were derived from the response to a total of 50 stimulus presentations. Statistical analyses are shown in Figures 2 and 3. **C**) Examples of averaged ERP waveform following 10 tone pairs **D**) 20 tone pairs **E**) 30 tone pairs and **F**) 40 tone pairs and **G**) 50 tone pairs.

Nicotine significantly increased amplitude of the P20 component and reduced amplitude of the N40, consistent with previous studies (Figure 1) [10], [11], [15], [40].

### P20 Amplitude

#### Experiment 1 (Nicotine antagonists DhβE and MLA)

**Figure 2:**
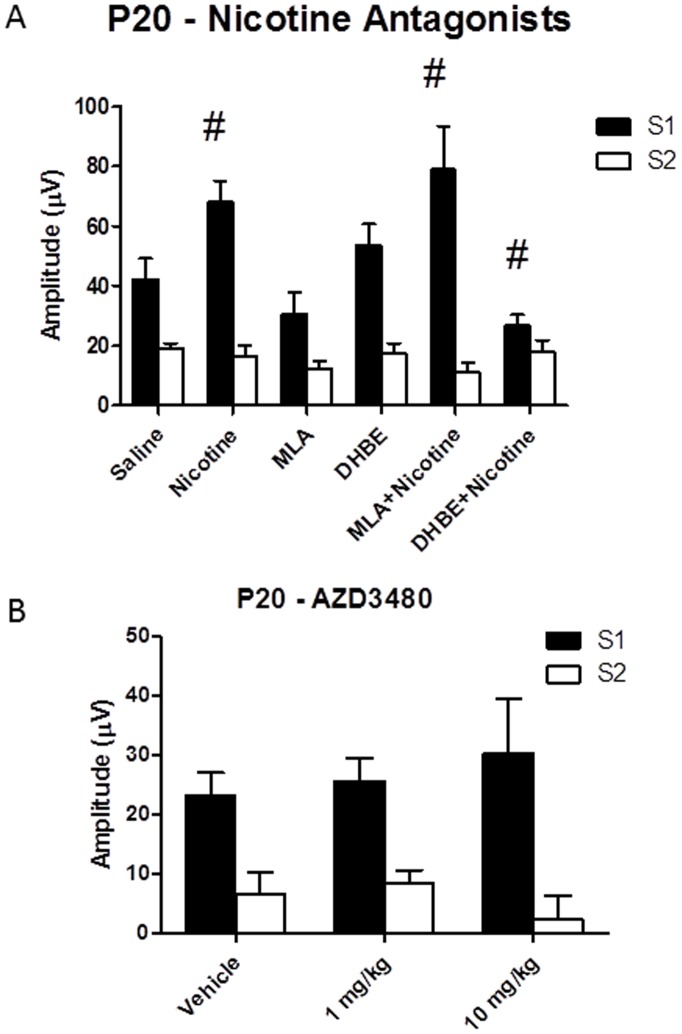
Nicotine receptor subtype-specific effects on the P20 component of the ERP. **A**) P20 amplitude following nicotine and nicotine receptor antagonists. Assessment of the effects of nicotine antagonists was carried out using a within subjects design in which each mouse was assessed on each drug, with sessions being separated by a 24 hour washout period. N = 13 for each condition. Nicotine (1 mg/kg) produced a significant increase in P20 S1 (black) amplitude without concomitant changes in S2 (gray). The α4β4/α4β2 nicotinic receptor antagonist DHβE (2 mg/kg) blocked the effect of nicotine on P20 amplitude, while the α7 antagonist MLA (10 mg/kg) had no effect on P20. S1 was significantly higher than S2 in all conditions except nicotine plus DHβE. **B**) **P20 amplitude following the α4β2 specific agonist AZD3480.** Assessment of the effects of AZD3480 was carried out using a within subjects design in which each mouse was assessed on each drug, with sessions being separated by a 72 hour washout period. The mice used to assess AZD3480 were a separate cohort from those shown in Figure 2A used to assess nicotine antagonists. N = 9 for each condition. No significant changes were observed on P20 amplitude following either a low (1 mg/kg) or high (10 mg/kg) dose of the α4β2 AZD3480 antagonist. # indicates significant difference from saline vehicle (all p<0.05).

*Increases in P20 amplitude following nicotine are blocked by pretreatment with DHβE, but not by MLA.* Assessment of the effects of nicotine antagonists was carried out using a within subjects design in which each mouse was assessed on each drug, with sessions being separated by a 24 hour washout period. N = 13. A repeated measures ANOVA on data from the pre-drug baseline trial of each session for the antagonist study revealed no significant differences over time [F(4,48) = 2.42, p>0.05], indicating that there were no significant lasting effects of any drug treatment. The mean amplitude and standard error of the mean (in parentheses) for each drug condition for S1 was; saline: 42.27 (6.84); nicotine: 67.98 (7.04); MLA: 30.65 (7.42); DHβE: 53.69 (7.01); MLA+nicotine: 78.96 (14.55) and DHβE+nicotine: 28.83 (3.55). Amplitude of the P20 component was significantly affected by drug treatment [F(5,60) = 6.33]. There was also a significant main effect for STIM [F(1,12) = 27.2], suggesting that significant sensory gating occurred, and for the STIM×DRUG interaction [F(5,60) = 11.38], suggesting that drug effects on P20 amplitude differed for the first and second stimuli. Post hoc testing on responses following S1 revealed that nicotine alone significantly increased P20 amplitude while neither MLA nor DHβE alone had a significant effect on P20 amplitude. The effect of Nicotine on P20 amplitude was blocked by pre-treatment with DHβE but not by pre-treatment with the α7 antagonist MLA. There were no significant treatment effects on P20 amplitude following S2 (Figure 1 and 2). *Comparison of S1 and S2:* The results of the statistical analyses were also used to draw inferences about sensory gating. Since sensory gating involves a reduction in S2 response relative to S1, a significant difference between S1 and S2 is suggestive of intact sensory gating. Post hoc comparisons from the analysis above revealed that nicotine, MLA- and DHβE-treated animals displayed normal P20 gating relative to saline vehicle treated controls. However, P20 gating was disrupted in animals treated with DHβE plus nicotine, (Figure 2), suggesting that this treatment combination disrupted sensory gating.

#### Experiment * (*AZD3480)

*Amplitude of the P20 component was not affected following treatment with the a4b2 agonist AZD3480*The mice used to assess AZD3480 were a separate cohort from those used to assess nicotine antagonists. N = 9. Assessment of the effects of AZD3480 was carried out using a within subjects design in which each mouse was assessed on each drug, with sessions being separated by a 72 hour washout period. A repeated measures ANOVA on data from the pre-drug baseline trial of each session no significant differences over time [F(2,16) = 0.06, p>0.05], indicating that there were no significant lasting effects of AZD3480 treatment across the duration of testing. Mean values for P20 amplitude were; Saline: 23.37 (3.7); 1 mg/kg AZD3480; 25.24 (3.8) and 10 mg/kg AZD3480∶30.31 (9.2). No significant effects were observed for either DRUG or for the STIM x DRUG interaction following AZD3480 treatment, although there was a significant main effect for STIM [F(1,8) = 56.6]. *Comparison of S1 and S2:* Post-hoc tests showed increased S1 amplitude relative to S2 in all treatment conditions, suggesting that AZD3480 did not affect P20 gating.

### N40 Amplitude

#### Experiment 1 (Nicotine antagonists DhβE and MLA)

**Figure 3:**
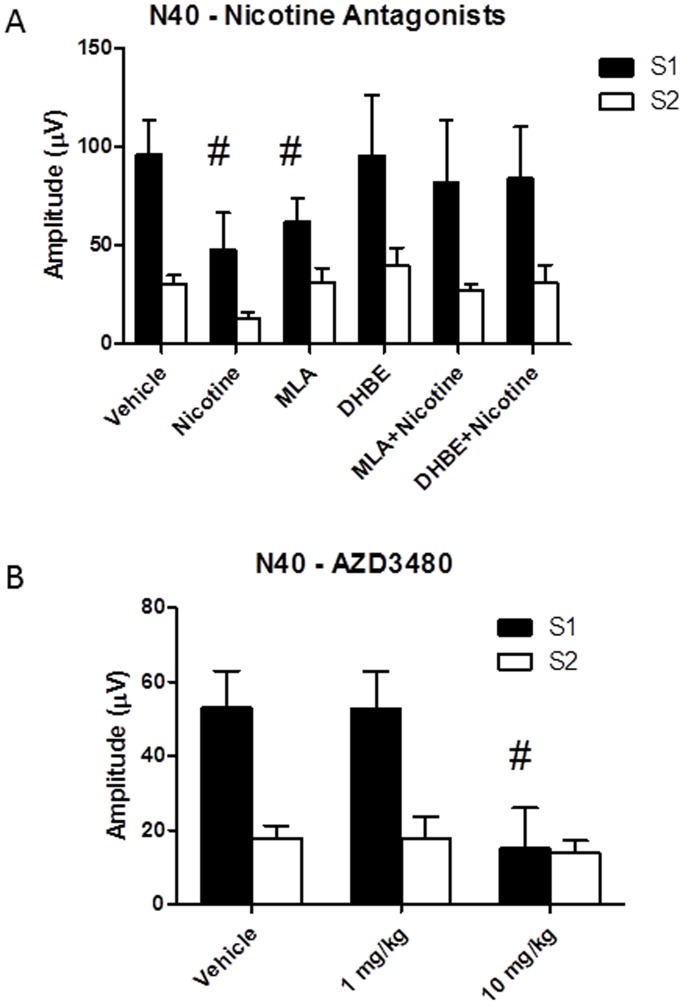
Nicotine receptor subtype-specific effects on the N40 component of the ERP. **A**) **N40 amplitude following nicotine and nicotine receptor antagonists.** Assessment of the effects of nicotine antagonists was carried out using a within subjects design in which each mouse was assessed on each drug, with sessions being separated by a 24 hour washout period. N = 13 for each condition. Nicotine (1 mg/kg) caused a significant reduction in the N40 ERP which was blocked by both DHβE (2 mg/kg) and MLA (10 mg/kg). These data suggest that activation of both α7 and α4β2 receptor systems are required for diminution of the N40 component. **B**) **N40 amplitude following the α4β2 specific agonist AZD3480.** Assessment of the effects of AZD3480 was carried out using a within subjects design in which each mouse was assessed on each drug, with sessions being separated by a 72 hour washout period. The mice used to assess AZD3480 were a separate cohort from those shown in Figure 2A used to assess nicotine antagonists. N = 9 for each condition. A significant reduction in N40 amplitude was seen following administration of the high dose (10 mg/kg) of AZD3480, suggesting that stimulation of the α4β2 receptor is sufficient to reproduce the effect of nicotine on this component. No significance was observed for the low (1 mg/kg) dose. No drugs tested showed any effect on S2 response - all significant effects were confined to changes in S1 amplitude. # - indicates significant difference from saline vehicle (all p<0.05).

*Decreases in N40 amplitude following nicotine are not blocked by pretreatment with MLA or DhβE. * A repeated measures ANOVA on data from the pre-drug trial of each session for the N40 component revealed no significant differences over time [F(4,48) = 1.0, p>0.05], indicating that the baseline N40 at each session was not affected by repeated testing or previous drug exposures. Amplitude of the N40 component was significantly affected by drug treatment [F(5,60) = 3.20] and by STIM [F(1,12) = 7.2]. There was no significant STIM×DRUG interaction, suggesting that drug effects on N40 amplitude did not differ for the first and second stimuli. Post hoc testing for the main effect of drug treatments revealed that nicotine significantly decreased N40 amplitude. A significant reduction in N40 amplitude was observed following MLA by itself. In contrast, DHβE alone did not alter N40 amplitude and neither antagonist blocked nicotine-induced decrement of N40 amplitude. The mean amplitude and standard error of the mean (in parentheses) for each drug condition for S1 was; saline: 95.87 (17.6); nicotine: 47.47 (18.9); MLA: 62.08 (11.9); DHβE: 95.46 (30.6); MLA+nicotine: 81.89 (31.2) and DHβE+nicotine: 83.84 (26.0). *Comparison of S1 versus S2:* A significant effect was observed for STIM [F(1,12) = 7.17], with N40 amplitude being significantly greater following S1 relative to S2. A lack of a significant STIM x DRUG interaction suggests that the difference between S1 and S2 was consistent across treatment conditions (Figure 3).

#### Experiment AZD3480

*Treatment with the α4β2 antagonist AZD3480 significantly reduced N40 amplitude.* A repeated measures ANOVA on data from the pre-drug trial of each session for the N40 component revealed no significant differences over time [F(2,16) = 1.88, p>0.05], indicating that the baseline N40 at each session was not affected by repeated AZD3480 treatment. Mean values for N40 amplitude following drug treatment were; Saline: 53.02 (9.9); 1 mg/kg AZD3480; 57.9 (9.9) and 10 mg/kg AZD3480∶15.04 (10.9). A significant effect was seen for DRUG [F(2,16) = 8.02], STIM [F(1,8) = 13.66], and the STIM x DRUG interaction [F(2,16) = 24.0]. Post hoc tests revealed significantly reduced S1 amplitude following treatment with the high (10 mg/kg) dose α4β2 agonist, relative to saline vehicle (Figure 3). *Comparison of S1 versus S2:* The α4β2 agonist produced a significant effect on gating of the N40 component, as revealed by a significant effect of STIM [F(1,8) = 13.66], as well as a significant interaction between STIM x DRUG [F(2,16) = 24.0]. Post hoc tests comparing S1 and S2 response as a function of drug condition revealed significant differences within the vehicle (p = 0.0001) and low dose (p = 0.0001) groups. In contrast, no difference was observed between S1 and S2 in the high dose group, suggesting disrupted gating. Further, significant differences in N40 amplitude between the high dose and vehicle groups was only seen for the S1 component (p = 0.0001), demonstrating that reduced gating was due solely to reduced S1 response (Figure 3).

### Baseline and Evoked Gamma

#### Experiment 1 (Nicotine antagonists DhβE and MLA.)

*Pretreatment with DHβE, but not MLA, blocked the ability for nicotine to enhance evoked gamma oscillations.* A repeated measures ANOVA on pre-drug baseline data for evoked gamma revealed no significant differences over time [F(4,48) = 0.11, p<0.05], indicating that the evoked gamma activity was not affected by repeated testing or previous drug exposure. Event-related gamma was significantly affected by drug treatment [F(5,60) = 6.31, p<0.05]. Post hoc tests showed enhanced gamma power following nicotine (p = 0.037) and following MLA + nicotine (p = 0.0002). No change was observed following DHβE treatment alone, while pretreatment with DHβE blocked the effect of nicotine on gamma power. MLA treatment by itself had no effect on gamma. Mean values for evoked-gamma were: Saline: 1.31 (0.28); Nicotine; 1.82 (0.29); MLA: 0.83 (0.33); DHβE: 1.7 (0.45); MLA+Nicotine: 2.39 (0.31) and DHβE+Nicotine: 1.41 (0.57). This pattern of results suggests that the effects of nicotine on event-related gamma are due to activation of the a4b2 receptor.

#### Experiment 2 AZD3480

**Figure 4:**
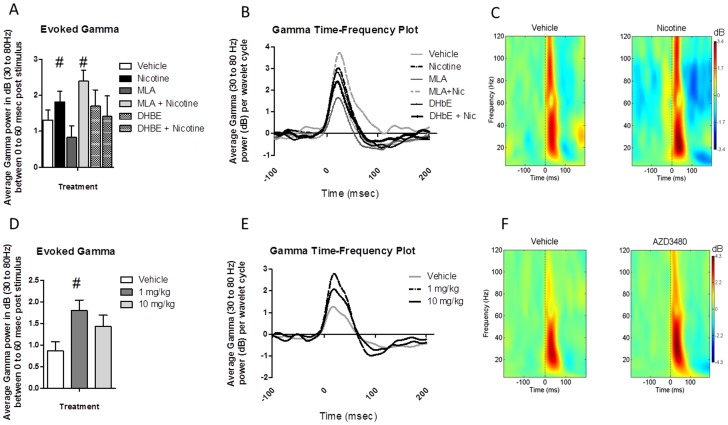
Nicotine receptor subtype-specific effects on auditory evoked gamma oscillations. (Top) Event-related Gamma (30 to 80 Hz) activity following nicotine and nicotine receptor antagonists. Assessment of the effects of nicotine antagonists was carried out using a within subjects design in which each mouse was assessed on each drug, with sessions being separated by a 24 hour washout period. N = 13 for each condition. Depicted top left **A**) is the average event-related gamma power (in dB) for the period between 0 and 60 msec following stimulus onset, across all 50 stimulus presentations. This was done by calculating the time-frequency response for each wavelet cycle within the 0 to 60 msec period and averaging the resulting values to create a single number. All statistical analyses were conducted on these values. Nicotine (1 mg/kg) caused an increase in evoked gamma activity, which was blocked by DHβE (2 mg/kg) but not MLA (10 mg/kg), indicating that the positive effect of nicotine on gamma activity is mediated through the α4β2 receptor subtype. # indicates p<0.05. Top center **B**) depicts event-related gamma power across each individual wavelet cycle between 100 msec pre-stimulus and 200 msec post-stimulus across all 50 stimulus presentations. Depicted top left **C**) is a heat map showing event-related power in decibels (event-related spectral perturbation, i.e. the power following the stimulus expressed as a change from baseline) from 200 msec prior to stimulus onset to 200 msec post onset (0 = stimulus onset) across all 50 stimulus presentations following vehicle and nicotine treatment. (Bottom) Event-related Gamma activity following administration of the α4β2 specific agonist AZD3480. Assessment of the effects of AZD3480 was carried out using a within subjects design in which each mouse was assessed on each drug, with sessions being separated by a 72 hour washout period. The mice used to assess AZD3480 were a separate cohort from those shown in Figure 2A used to assess nicotine antagonists. N = 9 for each condition. Bottom left D) shows a significant increase in event-related gamma following low (1 mg/kg), but not high (10 mg/kg) dose AZD3480. The methodology used to create bottom left **D**), center **E**) and right **F**) figures are the same used to create figures **A**, **B** and **C**, respectively. # - indicates significant differences from saline vehicle (all p<0.05).

*The a4b2 agonist AZD3480 significantly enhanced evoked-gamma oscillations.* A significant effect of AZD3480 was observed on event-related gamma [F(2,16) = 3.7]. Post hoc tests showed a significant increase in gamma power following low dose (p = 0.016), but not high dose (Figure 4). Mean values and standard error of the mean (in parentheses) for event-related gamma were; Saline: 0.86 (0.21); 1 mg/kg: 1.8 (0.24) and 10 mg/kg: 1.43 (0.26).

### Limitations

One limitation of this study was the within-subject design. Minimum washout periods of 24 hours were provided between each drug treatment and the half-lives in rodents of the drugs used in this study did not exceed 30 minutes [41], [42], [43]. In addition, we performed analyses of the pre-treatment data for each testing day to address the potential effects of time and repeated drug exposure for each dependent variable. As reported, there were no significant effects of repeated testing for any variables, suggesting that neither time nor exposure to multiple drugs affected baseline responses in this study.

## Conclusions

Consistent with previous reports [10], [11], [15], nicotine significantly increased amplitude of the P20 response to the first click of a paired-click stimulus (S1), but failed to alter response to the second (S2) click. Administration of the α4β4/α4β2 antagonist DHβE by itself did not affect P20 amplitude, but blocked nicotine-induced increases in P20 amplitude. In contrast, administration of the highly selective α7 antagonist MLA did not affect the P20 response when presented alone and failed to block nicotine induced enhancements of P20 amplitude. Taken as a whole, this pattern of data suggests that the ability for nicotine to enhance P20 amplitude occurs primarily through activation of a DHβE sensitive, and not α7 receptor subtype sensitive mechanism. In contrast to the effects of receptor specific antagonists, administration of the α4β2 agonist AZD3480 had no effect on P20 amplitude, consistent with previous findings in which the effect of nicotine on P20 amplitude was not disrupted in β2 knockout mice. DHβE has approximately 10 fold higher affinity at α4β4 receptors than at α4β2 [44], suggesting that the enhancing effects of nicotine on the mouse P20 are mediated by α4β4 receptors. The β4 receptor subunit plays a key role in mediating the rewarding and addicting effects of nicotine [45] and is expressed in brain regions that are likely important for the P50 response, such as the medial habenula. It should be noted that the effect of DHβE was not simply to block the effect of nicotine on P20 but rather produced a significant decrease in amplitude relative to saline vehicle or to DHβE treatment alone. This suggests that DHβE actually reversed the direction of the effect of nicotine on P20 amplitude and that this likely occurred through a mechanism other than that activated by DHβE treatment alone. While previous reports have suggested a role for α7 in regulating both P20 amplitude and gating in rodents [21], [22], [23], [46], the current study failed to provide evidence consistent with this notion. It is likely that there are multiple nicotinic receptor subtypes that mediate the effect of nicotine on P20 amplitude, including the α7 and β4 subtypes, and that inactivation of α7 activity alone is not sufficient to fully block the response to nicotine. Interestingly, a non-significant (p = 0.08) trend towards reduced P20 amplitude was observed following MLA treatment alone, suggesting that blockade of the α7 receptor in the absence of nicotine produced an effect opposite to that seen following agonist treatment, which would be consistent with the notion of a limited regulatory role of α7 in P20 amplitude. Also consistent with the findings on P20 amplitude, MLA did not disrupt any aspect of P20 gating, either when administered alone or prior to nicotine treatment. In contrast, DHβE + nicotine produced a significant disruption of P20 gating, primarily due to a reduction in S1 response. This suggests that the effect of nicotine on P20/P50 gating may occur through a DHβE sensitive mechanism.

Similar to previous reports, nicotine significantly decreased N40 amplitude [10], [11], [15]. Administration of the α4β2 agonist AZD3480 significantly reduced N40 amplitude in a manner consistent with that seen following nicotine treatment. Likewise pretreatment with DHβE blocked the ability for nicotine to attenuate N40 amplitude. This pattern of results is consistent with evidence that nicotine alters N40 response through activation of the β2 subunit [25]. A significant reduction in N40 was observed following MLA treatment alone, suggesting a possible role for α7 in mediating the N40 response. While this result is also consistent with the notion that blockade of the α7 receptor may have subsequently led to increased activation of the α4β2 receptor, MLA pretreatment was also sufficient to block the effect of nicotine on N40 response. Thus, stimulation of α7 receptor may play some role in regulating N40 amplitude. The N40, like the P20, displayed gating such that responses to S1 were significantly larger than responses to S2. However, unlike the P20, there was no interaction between stimulus and drug treatment, suggesting that N40 gating was not significantly affected by treatment with any of the antagonists used here. In contrast, AZD3480 significantly reduced gating, largely by reducing amplitude of the S1 component. These results suggest that the mechanisms that govern N40 gating are largely consistent with those that govern N40 amplitude and primarily involve stimulation of the β2 receptor.

Gamma activity has been associated with perceptual and cognitive processes as well as positive and negative symptoms in schizophrenia [47], [48], [49], [50], [51], [52]. In the present study, nicotine increased event-related gamma oscillations, replicating a previous study in our laboratory and a previous report regarding the effects of smoking on gamma [15], [16]. The nicotine-induced increases in evoked gamma were blocked by DHβE but not by MLA, suggesting a role for the α4β2 or α4β4 receptor in mediating these effects. Consistent with this interpretation, treatment with AZD3480 significantly increased both baseline FFT and event-related power within the gamma range, further suggesting that α4β2 receptors are critical to this effect. While there is much evidence to suggest therapeutic effects of nicotine on schizophrenia symptomology and cognitive function, few studies have assessed the effect of nicotine on gamma oscillations. The findings reported here raise the possibility that nicotine could produce some of its therapeutic effects through enhancement of gamma activity, and that this likely occurs primarily through stimulation of the β2 receptor subunit.
